# Phase Transitions and Electric Properties of PbBr_2_ under High Pressure: A First-Principles Study

**DOI:** 10.3390/ma15228222

**Published:** 2022-11-19

**Authors:** Lihua Yang, Yukai Zhang, Yanli Chen, Xin Zhong, Dandan Wang, Lin Fan, Jihui Lang, Xin Qu, Jinghai Yang

**Affiliations:** 1 Key Laboratory of Functional Materials Physics and Chemistry of the Ministry of Education, College of Physics, Jilin Normal University, Siping 136000, China; 2 State Key Laboratory of Integrated Optoelectronics, College of Materials Science and Engineering, Jilin University, Changchun 130012, China

**Keywords:** high pressure, structure prediction, simulation, structural evolution, lead halide

## Abstract

PbBr_2_ has recently attracted considerable attention as a precursor for lead halide perovskite-based devices because of its attractive properties. It is known that pressure can modify the chemical and physical properties of materials by altering the distance between atoms in the lattice. Here, a global structure-searching scheme was used to explore the high-pressure structures of PbBr_2_, whose structures and properties at high pressure are still far from clear. Three new phases of PbBr_2_ were predicted in the pressure range of 0–200 GPa, and the pressure-driven phase transition sequence of orthorhombic Pnma (0–52 GPa) → tetragonal I4/mmm (52–80 GPa) → orthorhombic Cmca (80–153.5 GPa) → orthorhombic Immm (153.5–200 GPa) is proposed. Electronic calculations indicate a semiconductor-to-metallic transition of PbBr_2_ in the Cmca phase at ~120 GPa. Our present results could be helpful in improving the understanding of fundamental physical properties and provide insights to modulate the structural and related photoelectric properties of PbBr_2_.

## 1. Introduction

Lead bromide (PbBr_2_) is drawing attention due to significant scientific and technological applications. The presence of Pb with a high effective atomic number (Z eff = 82) enables PbBr_2_ to have a superior absorption ability for high-energy photons, so that PbBr_2_ could be applied in X-ray or γ-ray detection [1,2,3]. Due to its good acousto-optic merit and wide transmission range, PbBr_2_ has been applied in acousto-optic devices [4,5,6,7]. The crystal growth, purification, and optical and acoustic character of PbBr_2_ have been studied over the past few decades [6,7,8,9,10]. Recently, PbBr_2_ has been used extensively to prepare perovskite-based devices, such as the hybrid perovskites solar cells [11,12,13], photodetectors [14], led light-based devices [15], photocatalysts of antibiotics [16] and luminescent complexes in solvents [17,18].

It is known that pressure can alter the chemical and physical properties of materials as it can adjust the distance between atoms in the lattice [19,20,21,22,23,24]. At ambient conditions, PbBr_2_ is a semiconductor material and crystallizes in a cotunnite phase (Pnma, Z = 4) similar to many other AB_2_ compounds [9,10,25,26]. Theoretical and experimental studies on the electronic properties and structural transition sequences under high pressure for PbBr_2_ are less investigated, hindering the in-depth exploration of the chemical and physical properties of PbBr_2_ under compression. Thus, it is essential to investigate the structural evolution of PbBr_2_ under pressure for the further design of lead halide perovskite photovoltaics.

In this work, we explored the structures of PbBr_2_ by the well-known CALYPSO structure searches method, combined with first-principles calculations at a wide pressure range of 0–200 GPa. Our results show that the pressure-driven structural evolution of PbBr_2_ is orthorhombic Pnma → tetragonal I4/mmm → orthorhombic Cmca → orthorhombic Immm phase with transition pressures of 52, 80 and 153.5 GPa, respectively. We further investigated the PbBr_2_ electronic properties under high pressure. Strikingly, the phase transition is accompanied by a semiconductor-to-metallic transition. Our results will advance the understanding of the structure and electronic properties of PbBr_2_ under extreme conditions.

## 2. Materials and Methods

Structure searches for PbBr_2_ were carried out by the CALYPSO method (by registering at http://www.calypso.cn, accessed on 5 June 2013) [27,28,29], which was verified successfully by many studies [30,31,32,33,34,35,36,37]. Each generation contained 50 structures, and the first generation was produced randomly with symmetry constraints. All structures were locally optimized using the VASP code [38]. Local optimizations performed during structure search were undertaken with the conjugate gradients method and were stopped when enthalpy changes became smaller than 1 × 10^−5^ eV per cell. Sixty percent of the lowest-enthalpy structures of each generation were used to produce the structures in the next generation by local Particle Swarm Optimization techniques, and the remaining 40% of structures were randomly generated within symmetry constraints to enhance structural diversity. During the structure searches, each newly generated structure was subjected to structural optimization at target pressures to obtain local-minimum configurations. All structure optimization, enthalpy and electronic properties calculations adopt the VASP code with the generalized gradient approximation functional (Perdew–Burke–Ernzerh) [39]. We employ the projector augmented wave [40] scheme to treat the valence electrons of Pb and Br as 5d^10^ 6s^2^ 6p^2^ and 3d^10^ 4s^2^ 4p^5^. A total of 1000–1200 structures were generated for each structure search calculation with an energy cutoff of 310 eV. To ensure convergence of the calculated data, a kinetic energy cutoff of 400 eV and dense k-point sampling with a grid spacing of 0.2 Å^−1^ were employed. We used the PHONOPY code to ensure dynamical stabilities of the predicted PbBr_2_ structures [25]. Electron localization functions (ELFs) were drawn using VESTA software [41].

## 3. Results and Discussion

**Figure 1:**
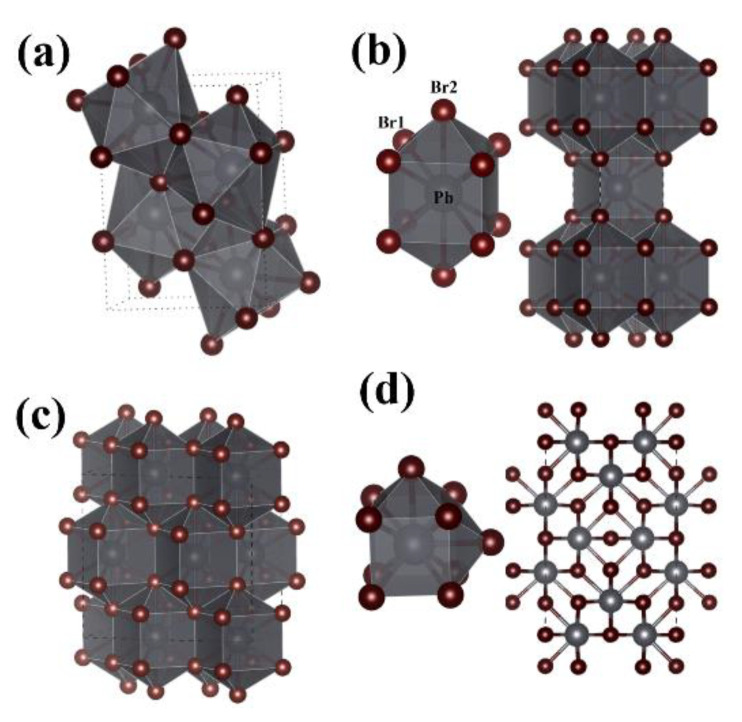
Crystal structures of stable PbBr_2_: (**a**) Pnma at 20 GPa, (**b**) I4/mmm at 60 Gpa, (**c**) Cmca at 100 Gpa, and (**d**) Immm at 200 GPa.

To discover stable structures of PbBr_2_ at high pressure, we executed systematic structural searches with 1–4 formula units at 0, 20, 50, 100, 150 and 200 GPa. According to our simulations, four energetically stable PbBr_2_ phases were found and are shown in Figure 1a–d. At 0 and 20 GPa, we reproduced the experimental Pnma structure (4 f.u., Z = 4). The unit-cell parameters of the predicted Pnma structure are very close to the experiment data [6,10], demonstrating the validity of the simulated method adopted here. As shown in Figure 1a, the Pb atom is ninefold coordinated by Br. Both Pb and Br atoms are located on the fourfold 4c site. Above 52 GPa, the already known PbBr_2_ with Pnma symmetry transforms into a tetragonal structure with I4/mmm (Z = 2) symmetry, accompanied by the nearest Br–Br distances shortened from 3.26 Å (at 20 GPa) to 2.88 Å (at 60 GPa). In the I4/mmm structure, Pb and Br atoms occupy the 2b site and 4e site, respectively. Within this structure, each Pb atom is tenfold coordinated by Br, forming a PbBr_10_ square prism with a double cap. At 60 GPa, the distance from the central Pb atom to the side Br1 atom and capped Br2 atoms of the prism is 2.836 Å and 3.022 Å, respectively. The Pnma and I4/mmm structures of PbBr_2_ are also predicted to exist in PbI_2_ and BaI_2_ [42,43].

**Figure 2:**
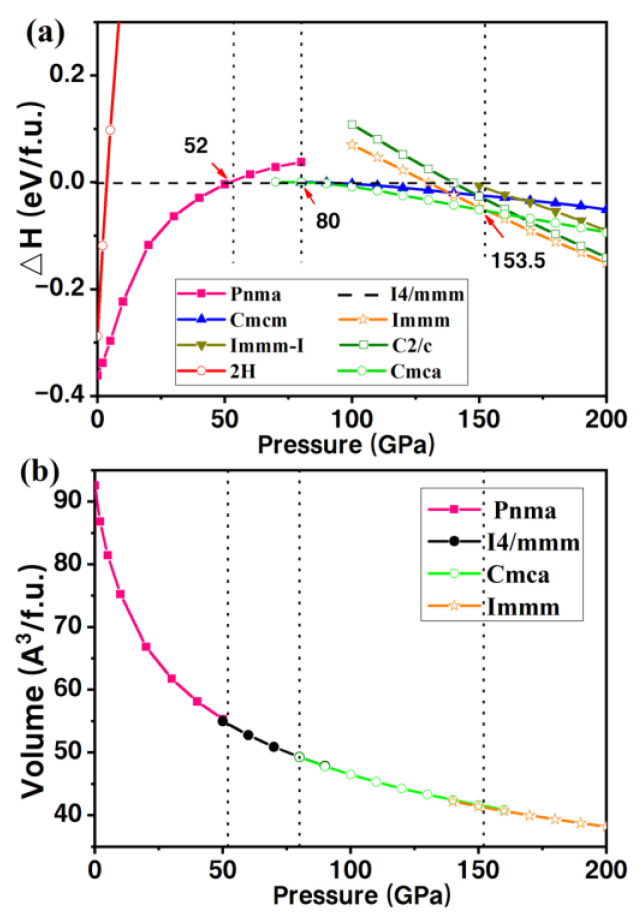
(**a**) Enthalpies related to the I4/mmm phase. (**b**) Relative volume of four stable PbBr_2_ phases.

Upon increasing the pressure to 80 GPa, PbBr_2_ adopts an orthorhombic Cmca structure with Z = 8. This structure can be seen as symmetry lowering of the I4/mmm structure. Within the Cmca structure (Figure 1c), Pb and Br atoms occupy the Wyckoff 8f site and 16g site, respectively, and the nearest Br–Br is 2.73 Å at 100 GPa. At a pressure above 153.5 GPa, an orthorhombic Immm (Z = 8) phase was found to be most stable up to 200 GPa. Significantly, the current PbBr_2_ Immm structure differs from the Immm structure found in PbI_2_ (Immm-I) [42]. While the former structure (Figure 1d) contains two distinct Pb atoms occupying the 4i and 4h sites, and four Br atoms occupying 2b, 2d, 4g, and 8l, respectively, the later has 2 f.u. in a unit cell with the Pb and I atoms occupying the Wyckoff 2d site and 4f site (the enthalpy difference of two Immm phases was calculated in Figure 2a). Within the Immm structure of PbBr_2_, the Pb atoms are tenfold coordinated, and the nearest Br–Br is 2.57 Å at 200 GPa. In conclusion, the Br–Br distances in the four stable PbBr_2_ structures are much smaller than those in pure Br_2_ (2.27 Å), indicating that no Br-Br covalent bond is formed.

**Figure 3:**
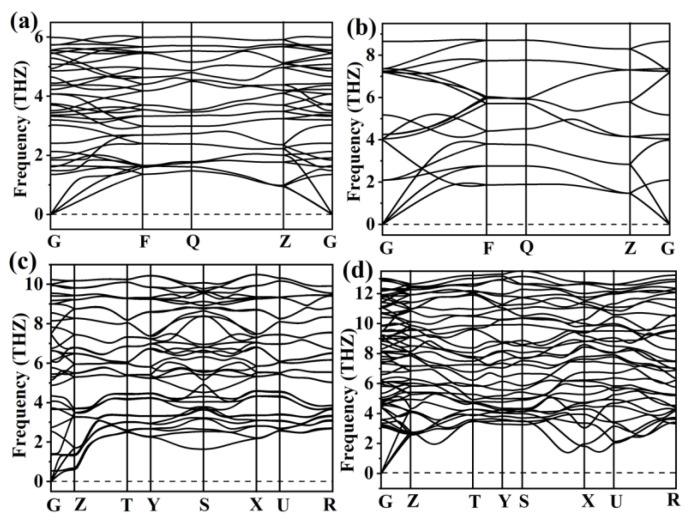
Phonon dispersion curves of stable PbBr_2_: (**a**) Pnma at 20 GPa, (**b**) I4/mmm at 60 GPa, (**c**) Cmca at 100 GPa, and (**d**) Immm at 200 GPa.

We calculated the enthalpy difference (ΔH) of four stable PbBr_2_ phases relative to I4/mmm PbBr_2_, as shown in Figure 2a. Several structures in a previous study on lead halide compounds [42] were also considered. Obviously, the four PbBr_2_ structures we searched have the lower enthalpies. The changes in volume as a function of pressure of the predicted PbBr_2_ are also plotted in Figure 2b. The continuous change of unit cell volume of PbBr_2_ with pressure suggests a second-order structural phase transition. Our work suggests that PbBr_2_ follows the structural transition order of Pnma (0–52 GPa) → I4/mmm (52–80 GPa) → Cmca (80–153.5 GPa) → Immm (153.5–200 GPa). Our phonon calculations demonstrate the dynamic stability of all these predicted PbBr_2_ structures, as shown in Figure 3. The detailed structural parameters and calculated phonon spectra of the predicted PbBr_2_ phases are shown in Table 1.

**Figure 4:**
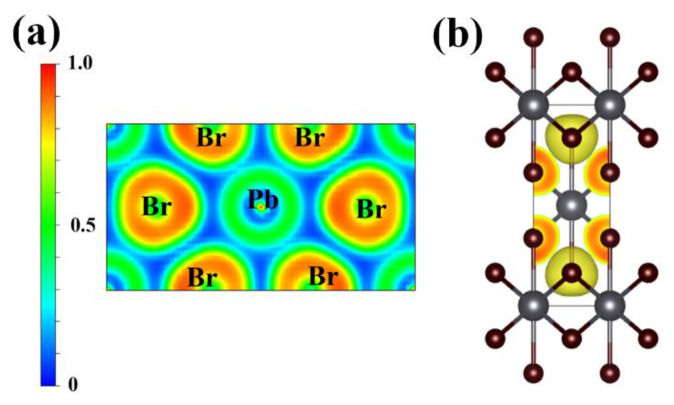
(**a**) 2D ELF plot through the (1–10) plane and (**b**) isosurface of the ELF plot with a value of 0.7 for I4/mmm PbBr_2_ at 60 GPa. Big spheres represent Pb atoms; small spheres denote Br atoms.

In order to further research the bonding properties of PbBr_2_ under compression, we investigated the ELF of four stable PbBr_2_ phases. All phases share similar features. We present the ELF of the I4/mmm phase in Figure 4 as a representative. The blue color corresponding to the ELF value < 0.5 is mostly around Pb atoms, indicating the electron shortage of Pb and electron transfer from Pb to Br. Because the ELF map does not show electron localization at the lattice gap, the bonding type between Pb and Br is mainly ionic. The isosurface of ELF with a value of 0.7 (Figure 4b) also indicates an electronic consumption near Pb atoms and cumulation around Br atoms, showing the charge transfer from Pb to Br, associated with the Pb–Br ionic bond.

**Figure 5:**
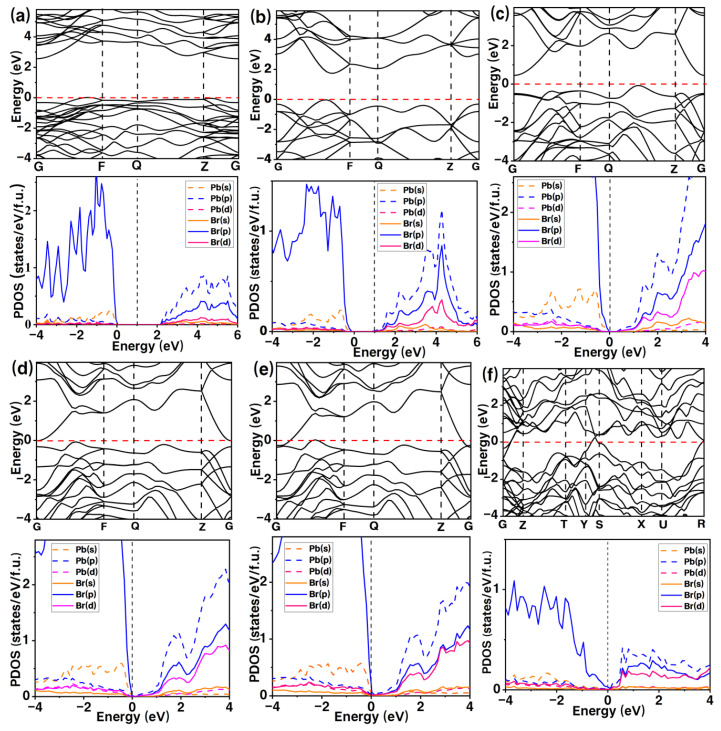
Calculated electronic band (**top**) and PDOS (**bottom**) plot of predicted PbBr_2_ phases: (**a**) Pnma at 20 GPa, (**b**) I4/mmm at 60 GPa, (**c**–**e**) Cmca at 80, 120, 140 GPa, and (**f**) Immm at 200 GPa, respectively.

We calculated the electronic band and partial density of states (PDOS) of the four predicted structures. The Pnma phase is a semiconductor with an indirect bandgap Eg = 2.11 eV, where the Pb-6p and Br-5p states dominate the valence band (VB) edge, and an anti-bonding hybridization of the Pb-6s and Br-5p states controls the conduction band (CB) edge, as shown in Figure 5a. The bonding character of the I4/mmm phase is similar to that in the Pnma structure, e.g., the band structure calculated at 60 GPa also shows an indirect band gap (Eg = 0.69 eV). The I4/mmm structure remains a semiconductor at the pressure stability interval (52–80 GPa). For the Cmca structure of PbBr_2_, the calculated band structures at 80, 120 and 140 GPa show that a semiconductor–metal transition occurs at ~120 GPa, because the CB extends across the Fermi level (Figure 5c–e). It can be seen that lead bromide metallizes at a higher pressure than lead iodide (~27 GPa). In the high-pressure Immm phase of PbBr_2_, both the VB and CB extend across the Fermi level, and the metallic nature of PbBr_2_ persists up to 200 GPa.

## 4. Conclusions

We studied the pressure-driven structural transformation and electronic properties of PbBr_2_ up to 200 GPa. The structural evolution of PbBr_2_ is orthorhombic Pnma → tetragonal I4/mmm → orthorhombic Cmca → orthorhombic Immm phase with transition pressures of 52, 80 and 153.5 GPa, respectively. Electronic calculations indicate that Pnma and I4/mmm phases are semiconductors, and a semiconductor-to-metallic transition of PbBr_2_ was found in the Cmca phase at ~120 GPa. The Immm phase maintains metallic properties throughout the pressure stabilization range. Our results will stimulate further studies on the behavior of AB_2_-type halides under extreme conditions.

## Figures and Tables

**Table 1:** Lattice parameters and atomic coordinates of a conventional unit cell of PbBr_2_.

Phase	Z	Lattice (Å)	Atom	X	Y	Z
Pnma 0 GPa	4	a = 8.04246 b = 4.76121 c = 9.66722	Pb(4c) Br1(4c) Br2(4c)	0.734 0.516 0.358	0.750 0.750 0.250	0.910 0.162 0.418
I4/mmm 60 GPa	2	a = b = 3.426 c = 8.993	Pb(2a) Br(4e)	0.000 0.000	0.000 0.000	0.000 0.336
Cmca 100 GPa	8	a = 8.548 b = 4.679 c = 9.295 α = β = γ = 90	Pb(8f) Br(16g)	0.000 −0.166	0.790 0.295	0.875 0.875
Immm 200 GPa	8	a = 3.598 b = 11.177 c = 7.594 α = β = γ = 90	Pb1(4i) Pb2(4h) Br1(2d) Br2(4g) Br3(8l) Br4(2b)	0.000 0.000 0.500 0.000 0.500 0.500	0.000 0.824 0.000 0.837 0.833 0.000	0.241 0.500 0.500 0.000 0.253 0.000

## Data Availability

The data presented in this study are available on request from the corresponding authors.
